# Predicting nursing students’ psychological well-being: network analysis based on a model of thriving through relationships

**DOI:** 10.1186/s12909-022-03517-1

**Published:** 2022-06-16

**Authors:** Lu Zhou, Khunanan Sukpasjaroen, YuMing Wu, Lei Wang, Thitinan Chankoson, EnLi Cai

**Affiliations:** 1 Chakrabongse Bhuvanarth International Institute for Interdisciplinary Studies, Rajamangala University of Technology Tawan-OK, Chonburi Bangkok, Thailand; 2 School of Nursing, Yunnan University of Chinese Medicine, Kunming, Yunnan China; 3 School of Medicine, Yunnan University of Chinese Medicine, Kunming, Yunnan China; 4 Faculty of Business Administration for Society, Srinakharinwirot University, Bangkok, Thailand

**Keywords:** Psychological well-being, Relationships, Nursing students, Network analysis

## Abstract

**Background:**

Psychological well-being plays a vital role in nursing students’ mental health and affects their decisions to stay in the nursing profession, particularly during the COVID-19 outbreak. Close relationships are undeniably linked to psychological well-being, but it is unknown how the specific pathways through which close relationships are related to each other and which are most strongly linked to nursing students’ psychological well-being.

**Aims:**

To explore the network structure, central and bridge factors among well-being characteristics, and predictors based on a model of thriving through relationships.

**Methods:**

A cross-sectional research design was used with a sample of undergraduate nursing students (531 participants from the Southwest part of China). We used a network model to analyze the network structure of perceived social support, mindfulness, self-integrity, self-compassion, professional self-concept, savoring, intentional self-regulation, non-relational self-expansion, relational self-expansion, attachment insecurity, and psychological well-being.

**Results:**

A highly interconnected network of psychological well-being featured predictors and traits were formed. Node 8 (self-kindness), node 9 (self-judgment), and node 23 (non-relational self-expansion) were the predictors with the highest centrality in the network. Perceived social support and professional self-concept were most central in linking predictors to psychological well-being traits. Attachment insecurity was a non-supportive factor for predicting psychological well-being among female nursing students.

**Conclusions:**

Interventions based on these supportive/non-supportive predictors, which operate on different psychological levels, hold promise to achieve positive effects on psychological well-being among nursing students.

**Supplementary Information:**

The online version contains supplementary material available at 10.1186/s12909-022-03517-1.

## Introduction

Psychological well-being(PWB)—is broadly defined as the “positive perception of engagement with different challenges of life” [1, 2], and it refers to one’s sense of growth and self‐realization, to how people efforts to facilitate experiences of purposeful engagement [3]. Research indicated that psychological well-being plays a vital role in influencing nursing students’ decision to enter and remain in the nursing field and successfully adapt to college/university life [4]. Unfortunately, their psychological well-being has been struck by COVID-19 badly [4, 5]. Moreover, nursing students as a group, globally, may be inherently more vulnerable to psychological distress [6]. In China, the prevalence of anxiety, depression, or comorbid anxiety and depression was 55.0%, 56.4%, and 31.6%, respectively [7]. Numerous calls for paying attention to social support would be an effective way to improve nursing students’ well-being [8–10].

The years of early adulthood, with key tasks of self-identity and developing close relationships, engender both opportunities and risks for thriving. Despite a wealth of evidence linking perceived social support to psychological well-being in clinical and non-clinical populations, how to promote psychological well-being via factors based on close relationships remains unknown, and most works in this area lack theoretical support. Feeney and Collins [11] proposed a theoretical model of thriving through relationships, and there are eight categories of pathways linking social support to well-being: emotional state, self-perceptions, appraisals of events, situation-relevant behaviors and resources, relational outcomes, motivational state, neural activation, and physiological functioning, and lifestyle behaviors. This model of thriving through relationships provided a theoretical foundation for identifying the specific interpersonal processes that underlie the effects of close relationships on thriving [11], extending attachment theory in which relational attitudes influenced by social support contribute to well-being outcomes.

The first six pathways (i.e., emotional state, self-perceptions, appraisals of events, situation relevant behaviors and resources, relational outcomes, and motivational state) in the Feeney and Collins [11] model exactly map on to mindfulness, self-integrity, self-compassion, professional self-concept, savoring, intentional self-regulation, relational self-expansion, and non-relational self-expansion. Attachment insecurity was added to the study. Additionally, there is a substantial amount of evidence linking each of these nine factors to psychological well-being.

The first pathway of the model demonstrated that social support allows for one's objective emotional states and promotes self-monitoring capacity and objective observability, which has positive effects on promoting intrinsic motivation and thriving due to focus on the present moment [12]. Mindfulness is a self-regulatory approach in which an individual awareness of the present moment rather than the experience without judgments [13, 14]. The mindful capability allows for more self-consistent strategies to deal with undesirable stimuli and distress [14]. Several types of research linked mindfulness to better psychological well-being [15].

According to the second pathway (i.e. self-perception/self-acceptance), we chose three predictors (i.e. self-compassion, self-integrity, and professional self-concept). Self-compassion, i.e., benevolence and concern for oneself. [16]. According to cognitive neuroscience, self-compassion also associates with the soothing system of the emotion regulation system and therefore promotes well-being [17]. In addition, differences due to nursing and non-medical backgrounds may alter the perception or experience of self-compassion. There is a greater emphasis on compassion in nursing cultures [18]. Self-Integrity is defined as a process and tendency that individuals build connections between differentiating and multiplying self components, increase the level of conceptualization, and eventually form a sustainable and stable core self [19]. Individuals with self-affirmative resources are less reactive to and defensive about stressors because their overall sense of self-integrity relies less on the outcome of that particular stressor [20–23]. Research studies on self-concept differentiation, in contrast to self-integrity, have found that self-concept differentiation is associated with negative mental health outcomes, such as anxiety [24], loneliness [25] and depression [25, 26]. As with self-compassion, professional self-concept is also a type of self-representation that encompasses self-perception and self-evaluation through nursing activities, knowledge, and interpersonal relationships in the surrounding environment [27]. Limited research has shown that negative self-concept of university nursing students is associated with depression and anxiety [28].

Compared to non-medical students, nursing students may encounter more challenges in their life, such as death, negative professional image, workplace violence, etc. [8]. Greater ability to focus on positive experiences is the key to overcoming obstacles and challenges and predicted greater Psychological Well-Being [29, 30]. According to Bryant and Veroff (2007), savoring is the capacity to regulate your positive feelings by directing your attention to positive experiences, appreciating these experiences, and elaborating or enhancing your experience of these positive moments in one’s life [29, 31, 32].

Intentional self-regulation(ISR)-conceptualized by the SOC model of Selection, Optimization, and Compensation-is an essential skill in promoting long-term thriving and adaptive development across the life span [33]. This kind of ISR can deal with goal conflict and reach goals [34]. Those higher in goal conflict report lower well-being (e.g. [35, 36]). There are studies linking SOC strategies to better subjective well-being [37], as well as psychological well-being [38]. According to Feeney and Collins [11], social support also leads to better self-regulation and coping strategies that the ability to control one’s behavior, emotions, and thoughts [39].

Relational self-expansion(RSE)-refers to a relational attitude that which a person adopts the attributes of a partner into one’s self-concept in a close relationship [40]. Relational self-expansion will be independent of negative life events and emotional distress [41], and will enhance relationship satisfaction and one's potential [42]. Thus, this process can promote one’s self-development and positive perceptions. Several kinds of research indicated that self-expansion has an overall positive effect on well-being [43–45].

Non-relational self-expansion (NRSE)-is defined as an intrinsic motivation to enhance one’s self-efficacy, which occurs by engaging in novel, challenging, and exciting activities, and incorporating others perspectives and experiences into their self-concept [46]. Self-expansion is positively associated with a variety of well-being outcomes (e.g. life satisfaction, positive affect, and psychological well-being) for both individuals [47] and their relationships [48], as well as decreased depression and perceived stress [46]. It has been suggested that social support may promote motivation (self-expansion) by allowing boldness and willingness to pull up stakes [11].

Attachment is typically conceptualized based on two dimensions: anxiety and avoidance [49]. Researches that link attachment to well-being are relatively recent and have focused more on SWB than on PWB. Limited literature has explored the relation between attachment insecurity and psychological distress [50].

Even though the supportive and non-supportive factors listed above are well supported by the theory of thriving through relationships and previous studies, interactions between different predictors and well-being traits remain unclear. Network analysis is well-suited to model these relations because of the complexity that may be involved. Network analysis is a statistical and theoretical framework that has been utilized increasingly to explore the relations between a wide variety of disorders and symptoms in psychiatry [51]. By putting all factors into a network, the network analysis can estimate which factor is most “central” or “bridged”. A drawback of previous studies is that for the most part, they have only focused solely on single pathways and not on the interactions of a line of factors [52]. Limited study has explored how factors based on close relationships are related to nursing students’ traits of psychological well-being.

In the present study, we used network analytic techniques to better understand the interactive relations among traits and predictors of psychological well-being in nursing students, as well as to identify the central and bridged predictors.

## Methods

### Setting and participants

This study took place in southwest China, and 531 undergraduate nursing students 18–25 years of age (female: 71.8%, 2.9% freshmen, 3.5% sophomores, 6.6% juniors, and 86.8% seniors) from a medical in China (All full-time) took part.

### Study design and procedures

This is a cross-sectional study conducted between May 1, 2021, and September 1, 2021. A non-probabilistic sample of undergraduate nursing students was recruited. To increase response rates, the majority of the surveys were conducted during class time. Further, snowball sampling and posters and social media posts on a university campus were used to recruit undergraduate nursing students. Prospective participants were sent an email containing a link to a description of the study and a 30-min survey. Participants provided informed consent and completed an online pre-screening questionnaire for the real study, thereby confirming their identities. The researcher conducted an audit trail to ensure participants' mental health. In addition, the research team provided psychological counseling on a pro bono basis when needed by the participants to avoid potential stress caused by participation in the research. Participants received an extra reward for their time.

### Measures

#### The Multidimensional Perceived Social Support Scale (MSPSS)

Perceived social support was assessed using the 12-item Chinese version of the MSPSS [53]. Participants rated each item on a scale from 1 (very strongly disagree) to 7 (very strongly agree). We used a sum score of the full scale as an indicator. Higher overall scores indicated greater perceived social support. The scale has strong internal reliability in the samples used in this study (Cronbach’s α_Total_ = 0.84).

#### The Cognitive and Affective Mindfulness Scale-Revised (CAMS-R)

Mindfulness was assessed using the 12-item Chinese version of the CAMS-R composed of four primary scales measuring attention, present focus, awareness, and acceptance [54, 55]. Participants rated each item on a scale from 1 (rarely/not at all) to 4 (almost always). Higher scores indicated greater mindfulness. Cronbach’s alpha for the present sample was adequate (Cronbach’s a = 0.79 ~ 0.88).

#### The Self-Integrity Scale (SIS)

Self-Integrity was assessed using the 8-item Chinese version of the SIS composed of two primary scales measuring adaptive adequacy, and feelings of moral [56]. Participants indicated their agreement with each item on a scale from 1 (strongly disagree) to 7 (strongly agree). Higher scores indicated greater self-integrity. Cronbach's alphas were 0.84 ~ 0.91.

#### The Self-Compassion Scale (SCS)

Self-Compassion was assessed using the 26-item Chinese version of the SCS composed of six primary scales measuring self-kindness, self-judgment, common humanity, isolation, mindfulness, and over-identification [16]. Response option ranges from 1 (never do that) to 5 (always do that). The negative items were reversed scored, and the sum across all items was calculated. Higher scores indicated greater self-compassion. Cronbach's alphas were 0.77 ~ 0.89. Response option ranges from 1 (never do that) to 5 (always do that). The negative items were reversed scored, and the sum across all items was calculated. Higher scores indicated greater self-compassion [57]. Cronbach's alphas were 0.77 ~ 0.89. Note that: given that nursing students are in a high-stress environment, we followed Neff's [16] 6-component model, but recent studies suggested that omitting the 3 “negative” dimensions to measure self-compassion may be closer to its definition [58–60]

#### The Professional Self-Concept Scale (NSCI)

Professional self-concept was assessed using the 14-item Chinese version of the NSCI [27]. Response option ranges from 1 (definitely false) to 8 (definitely true). For the aim of our study, we used a sum score of the full scale as an indicator. Higher overall scores indicated a more positive professional self-concept. Cronbach's alpha was 0.92.

#### The Savoring Beliefs Inventory (SBI)

Savoring was assessed using the 24-item Chinese version of the SBI composed of three primary scales measuring anticipating, savoring the moment, and reminiscing [31], ranging from 1 (strongly disagree) to 7 (strongly agree). The 12 odd-numbered items were reverse-scored, and the mean of all items was calculated. Higher scores reflected a greater perceived ability to savor. Cronbach’s α alphas were 0.85 ~ 0.90.

#### The Measurement of Selection, Optimization, and Compensation (SOC)

The intentional self-regulation was assessed using the 12-item Chinese version of SOC composed of four primary scales measuring elective selection, loss-based selection, optimization, and compensation [33]. Each response was scored as 1 (SOC-related strategy) or 0 (non-SOC-related strategy). Higher scores indicated that the students’ intentional self-regulation can promote the maximization of the adaptive integration of changes in the self and context. Cronbach’s alphas were 0.69 ~ 0.82.

#### The Self-Expansion Scale (SES)

Relational self-expansion was assessed using the 14-item Chinese version of the SES [61]. Participants rated each item on a scale from 1 (not very much) to 7 (very much). Higher scores correspond to greater evaluations of relational self-expansion. Cronbach’s α was 0.88.

#### The Self-Expansion Preference Scale (SEPS)

Non-relational self-expansion was assessed using the 24-item Chinese version of the SEPS [46]. Participants rated each item on a scale from 1 (strongly disagree) to 7 (strongly agree). The 12 Self-Conservation items were reverse-scored. Higher scores correspond to greater evaluations of self-expansion. We used a sum score of the full scale as an indicator. Cronbach’s α was 0.89.

#### The Experiences in Close Relationships–Relationship Structures Scale (ECR-RS)

Attachment insecurity was assessed using the 9-item Chinese version of the ECR-RS composed of two primary scales measuring anxiety and avoidance [62], ranging from 1 (strongly disagree) to 7 (strongly agree). Item-2, 4, 6 & 9 were reverse coded. Higher overall scores indicated a more serious extent to participants feeling anxious about being abandoned and uncomfortable with intimacy and avoiding others. Cronbach’s α alphas were 0.82 and 0.73.

#### The Ryff’s Scale of Psychological Well-Being (RPWBS)

Psychological Well-being was assessed using the 18-item validated version of the RPWBS composed of six primary scales measuring positive relations with others, autonomy environmental mastery, personal growth, purpose in life, and self-acceptance [63, 64], ranging from 1 (strongly disagree) to 6 (strongly agree). Higher scores reflected better psychological well-being. Cronbach’s α alphas were 0.70 ~ 0.93.

#### Data analyses

A psychology network was constructed conducting in R (Version 3.3.0) to address the relations among the study variables. The package we used included qgraph [65], glasso [66], mgm [67], igraph [68], Exploratory Graph Analysis [69], and bootnet [70].

The score of 31 variables was used to estimate a Gaussian graphical model (GGM). variables (i.e. nodes) are bound together by conditional dependence relations (i.e. edges) in a network [70]. Thresholded EBICglasso utilizes the “least absolute shrinkage and selection operator”, which was used to reduce the edge estimates (i.e. avoid spurious edges) to estimate GGM [70]. The Fruchterman-Reingold algorithm was used to determine the network, according to the connections between nodes [71].

Betweenness, closeness, and strength were used to estimate the centrality of the network. The node strength was the priority to identify the central nodes due to the reliability, presenting the estimates of betweenness and closeness in Supplementary Fig. S1 [70]. Predictability is shown by the degree of filling to which the circle around each node [72], reflecting the proportion of variance in that node that can be explained by variance in the nodes to which it is connected [70, 73]

We used the bridge function of the Networktools package to identify bridge variables between predictors and psychological well-being in the network. By calculating bridge strength, we identified which predictor was most strongly connected to nursing students’ psychological well-being [74].

The NetworkComparisonTest(NCT) was used to test for differences in centrality for each node, overall connectivity, and network structure in order to identify the network differences between males and females [75]. The correlation stability (CS) coefficient reflects the stability of the parameters. A CS coefficient of 0.25 or above is recommended [70].

## Results

### The psychological well-being network

**Fig. 1:**
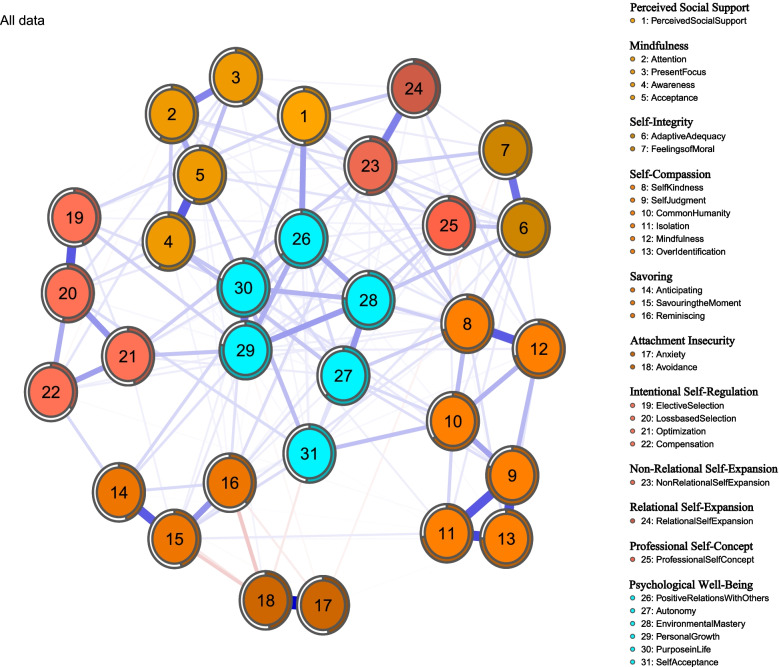
Network structure for predictors and traits of psychological well-being. Notes. Each variable is represented by a node (1 to 31), and it belongs to different variables, indicated by a code in the column on the right. Edges are represented by lines between nodes. Blue edges depict a positive association; red edges a negative association. Wider and more saturated edges reflect stronger associations. Predictability (i.e. the proportion of variance explained for a specific node by variance in nodes to which it is connected) is depicted as a filled part of a circle surrounding each node. The thickness of the line represents the connection strength. According to [73]

The psychological network structure is detailed in Fig. 1. Almost nodes within the network are related to each other positively except for attachment insecurity. The mean predictability was 0.562, which means that on average items from the model share 56.2% of the variance with surrounding items, demonstrating that there is 56.2% variation of nodes explained via variation among other nodes. We can know how well the given node can be predicted by the other nodes surrounding it assuming that all edges go to the node under investigation from its neighbors.

### Central nodes

**Fig. 2:**
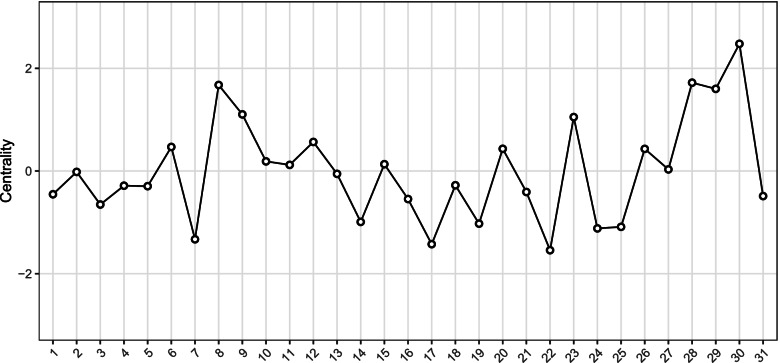
Strength centrality estimates for the 31 nodes. The Y-axis represents the centrality indices as standardized z-scores (the greater the estimate, the more central the item is), and the X-axis represents the 31 predictors and traits. According to [73]

Strength centrality for each node, that is, an estimate reflecting what degree each node was connected to other nodes in the network, is presented in Fig. 2. Centrality indices suggested that node 8 (self-kindness), node 9 (self-judgment), and node 23 (non-relational self-expansion) were central nodes in these specific pathways, and node 30 (purpose in life) was the central node in the psychometric measurement of psychological well-being. Node 22 and node 17 emerged as the least central nodes.

### Bridge nodes

**Fig. 3:**
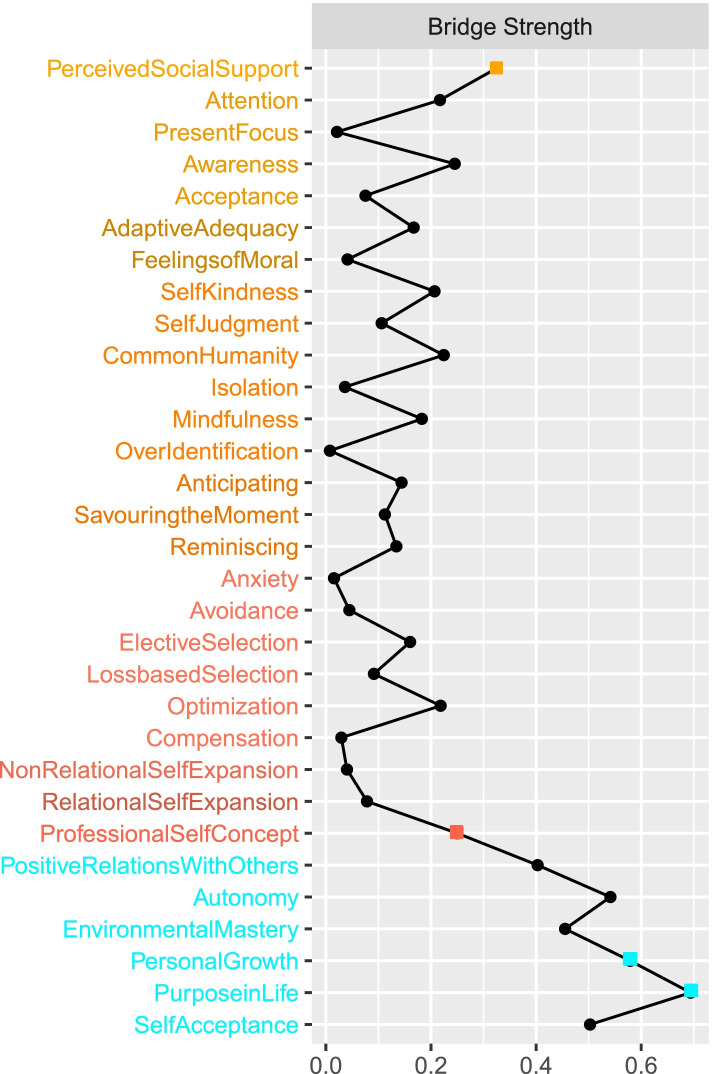
Bridge strength for each node. Notes. Bridge strength is a measure that reflects the degree to which each node links risk/protective factors and psychopathology symptoms. Z-standardised estimates are presented. Higher values indicate that a node is more important in linking predictors and traits of psychological well-being. According to [73]

To examine which nodes were most important in linking nursing students’ psychological well-being to predictors (i.e. the specific pathways through close relationships), we estimated bridge strength for each node. Results indicated that perceived social support and professional self-concept were most central in linking predictors to psychological well-being characteristics. All bridge strength centrality estimates are detailed in Fig. 3.

### Network accuracy and stability

The result of 95% confidence intervals of all edge weights indicated a good accuracy (detailed in Supplementary Fig. S2), and the network was identified accurately via the edge-weight difference test (detailed in Supplementary Fig. S3). CS coefficient values of strength, betweenness, and closeness are 0.75, 0.60, and 0.67, respectively. That is, the psychological well-being network was stable in the differences in node centrality (detailed in Supplementary Fig. S4). The centrality difference test showed that the highest centrality estimates are statistically different from the lowest centrality estimates (detailed in Supplementary Fig. S5).

There were no differences between males NS and females NS on the degree of overall interconnectedness of the network and the centrality of each node (*p* > 0.05). However, there were differences in network structure. Specifically, attachment insecurity was more strongly linked to relational self-expansion, savoring and positive relations with others among females than males (*p* < *0.01*), that is, these three nodes were significantly demonstrated more negatively associated among females than males.

## Discussion

By applying a network analytical approach, the study explored how the predictors based on the model of thriving through relationships (i.e. perceived social support, mindfulness, self-integrity, self-compassion, professional self-concept, savoring, intentional self-regulation, non-relational self-expansion, relational self-expansion, and attachment insecurity) were interrelated, and how these factors were related to psychological well-being in a sample of nursing students. The centrality of the network is stable and identified by a bootstrap approach. Furthermore, network comparison between gender provided evidence that the network has relatively high stability and generalizability. Overall, the results revealed a closely related network between predictors and characteristics of psychological well-being. A detailed description of the breakdown of each predictor is omitted here, due to restrictions on paper length, but each central node and bridge node is discussed individually.

In addition to the supportive predictors, attachment insecurity was significantly and negatively associated with both savoring beliefs and the self-acceptance dimension of psychological well-being among nursing students, suggesting that attachment insecurity was a non-supportive predictor for nursing students’ psychological well-being. Echoing previous theories suggesting that attachment insecurity may interfere with the improvement of the savoring ability and psychological well-being [76, 77]. According to attachment theory [78], individuals higher in attachment anxiety exhibit hyper-activation of the attachment system. Nursing students whose higher scores in both anxiety and avoidance may be consumed by insecurity and pay more attention to their emotions and feelings, thus weakening their strategies for savoring and self-acceptance [79–81].

Critically, the predictors that demonstrated the highest centrality were identified, which might be the measures and targets that can maximize the effectiveness of future interventions [82]. The node strength was used as the main centrality index because of its stability. Among all of the predictors, the nodes with the highest centrality are node 8 (self-kindness), node 9 (self-judgment), and node 23 (non-relational self-expansion). That is, the self-related attitudes play a vital role in influencing the other predictors, as well as psychological well-being traits among nursing students [57]. These results confirmed and extended previous research. First, in terms of self-compassion, these results are consistent with the previous study, indicating that lack of self-kindness may be an essential reason for nurses’ psychological distress in a large sample of nurses/nursing students in New Zealand [18]. It is true that self-compassion also avoids comparison with others or focuses on self-kindness/judgment, thus minimizing the distortion of self-concepts [83]. Self-compassion strengthens or facilitates the association between positive psychological constructs and both mental (e.g. [52, 84]), and physical (e.g., [85]) health outcomes. It suggests that the self-kindness and self-judgment dimensions of self-compassion are central ties of well-being traits and predictors, and this result that self-compassion has more positive effects on nurses group than doctors or medical students [18] seems to be cross-culture stable. Second, in terms of non-relational self-expansion, consistent with previous research, NRSE was associated with better psychological well-being, despite limited research that has explored the relationship between NRSE and other mental health outcomes [46]. This result was also validated in nursing students. By self-expansion, nursing students are more likely to increase self-efficacy and self-concept clarity to enhance nursing students' professional self-concept and professional identity due to the personal potential enhancement [46], thereby improving self-acceptance and self-growth to promote psychological well-being [11]. The relatively high stability (centrality) of node 8 (self-kindness), and node 9 (self-judgment) in our results suggest the importance of self-compassion in Chinese nursing students’ psychological well-being.

Interestingly, bridge strength indices suggested that perceived social support was most central in linking predictors to psychological well-being traits. These findings are generally consistent with a study by Feeney and Collins' theoretical model [12] that identified close relationships(e.g. social support) as the strongest protective factor for long-term thriving(e.g. Psychological well-being, social well-being, and physical well-being) in the general population, and even though in patients, and with the empirical study in the sample of college students in U.S.A [52], identifying that perceived social support predict well-being via the mediators of mindfulness, savoring and self-compassion. However, classical regression analysis was unable to reveal the complex variable network achieved in this study which may imply that perceived social support not only influences the rest of the predictors but can also be influenced inversely [86]. In addition, nursing students' professional self-concept was the central bridge node second only to perceived social support. the result may imply that the other predictors were partly linked to psychological well-being traits through professional self-concept, that is, the link between the rest of the predictors and well-being traits seems to be mediated by professional self-concept [57, 87]. The protective factor related close relationships seems to be promoted self-acceptance and self-identify, which are complementary to the structure of psychological well-being.

Finally, considering network differences between males and females, attachment insecurity has negative effects on relational self-expansion, savoring, and positive relations with others among females than males [88]. Research has found gender differences in attachment, showing that females tend to be significantly more attached to their peers than males [74, 89]. Similarly, in an Asian context, male college students showed higher trust toward close relationships when compared to female college students, whereas females showed higher levels of alienation [74]. Our results suggest that attachment insecurity was a non-supportive factor in predicting psychological well-being among female nursing students.

### Limitations and future research

Despite this being the first empirical study to reveal the complex variable network based on the model of thriving through relationships, it is also important to acknowledge the limitations of the current study. First, the data in the present study were obtained from self-reports, which can produce self-report response bias [90]. Second, it prevents inferences about how variables are causally related as all data were collected at a single time point. Future researchers are advised to explore the causal relationship among these examined variables in a longitudinal design [91]. Third, the generalisability of the findings is limited due to professional differences and sample characteristics. This means that network properties such as central nodes and bridge nodes may be unlikely to replicate in the same way in other medical groups [92]. Finally, despite our data providing evidence of predictors, there are undoubtedly additional supportive/ non-supportive predictors that contribute to and may also account for promoting better psychological well-being. Future research should replicate these findings, extending our results by exploring other pathways and research methods. Moreover, the findings revealed some core predictors, which may contribute to strategies for exploring intervention measures for nursing students' psychological well-being.

## Conclusions

The current study reveals that three predictors have the highest degree of centrality in nursing students’ psychological well-being network: self-kindness, self-judgment, and non-relational self-expansion, respectively. Perceived social support and professional self-concept were most central in linking predictors to psychological well-being traits. These predictors might serve as targets of interventions to promote nursing students' psychological well-being.

## Supplementary Information

**Additional file 1:** Supplementary file.

## Data Availability

The datasets analyzed in this manuscript are not publicly available. Requests to access the datasets should be directed to: zhoulutg@gmail.com.
