# Supplementary material for: Predicting nursing students’ psychological well-being: network analysis based on a model of thriving through relationships
Source: BMC Med Educ. 2022 Jun 16;22:463. doi: 10.1186/s12909-022-03517-1 (PMC9202322; doi:10.1186/s12909-022-03517-1)
Supplement: Supplementary file 1 — Additional file 1: Supplementary file. [file 12909_2022_3517_MOESM1_ESM.docx]

**Supplementary materials for:**

**Predicting Nursing Students’ Psychological Well-Being: Network Analysis Based on a Model of Thriving Through Relationships**

Lu Zhou [PhD candidate](https://onlinelibrary.wiley.com/action/doSearch?ContribAuthorRaw=Zhang,+Shan).^1,2^, Khunanan Sukpasjaroen.^1^, YuMing Wu.^3^, Lei Wang.^2^ , Thitinan Chankoson.^1,4^ *, and EnLi Cai^2.^*

1 Chakrabongse Bhuvanarth International Institute for Interdisciplinary Studies, Rajamangala University of Technology Tawan-OK, Thailand;

2 School of Nursing Yunnan University of Chinese Medicine, China;

3 School of Medicine, Yunnan University of Chinese Medicine, China;

4 Faculty of Business Administration for Society, Srinakharinwirot University, Thailand;

* Correspondence: luzhouynutcm@yahoo.com(T.C.); caienliycm@sina.cn(EL.C.)


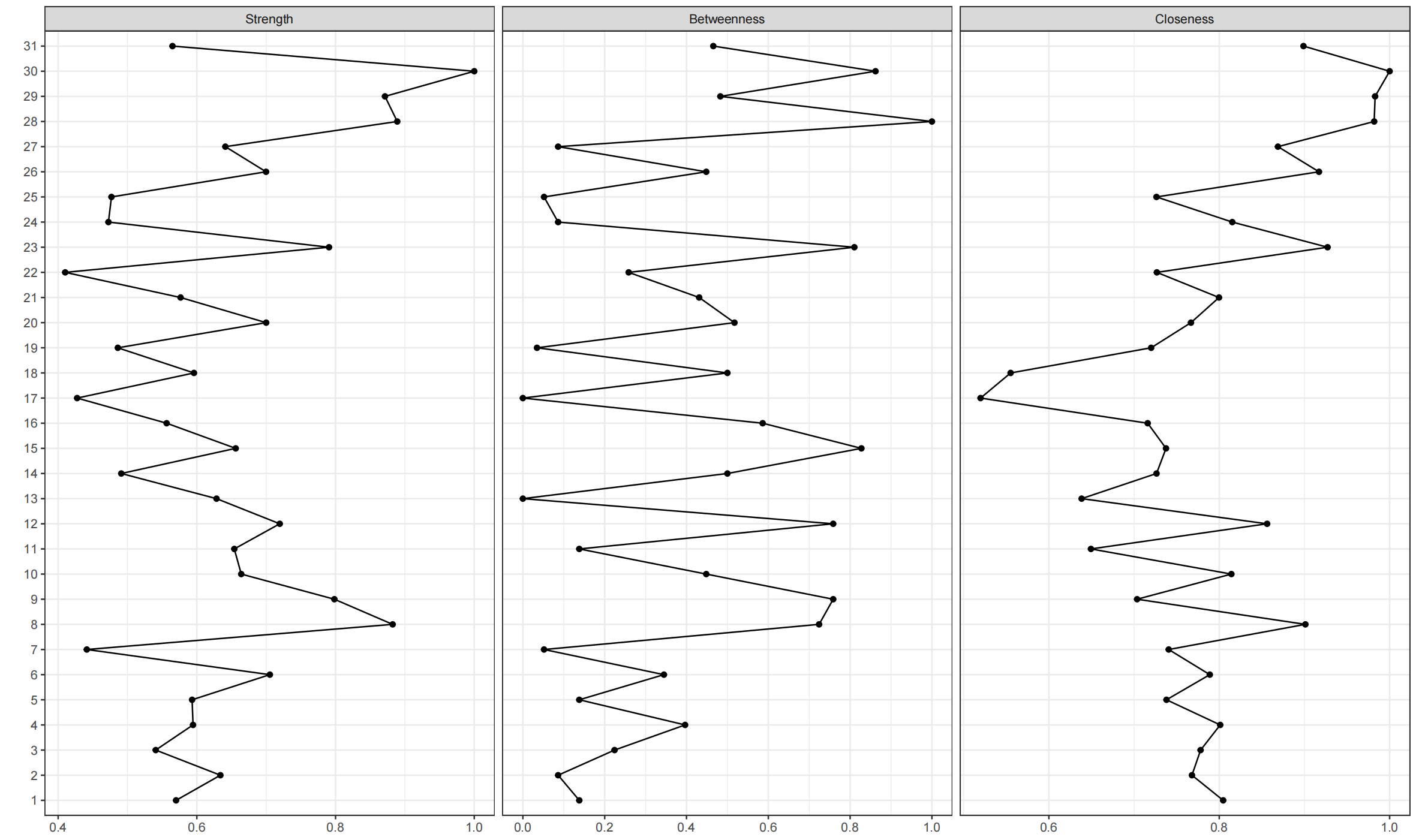


**Fig. S1** Strength, closeness and betweenness of psychological well-being network


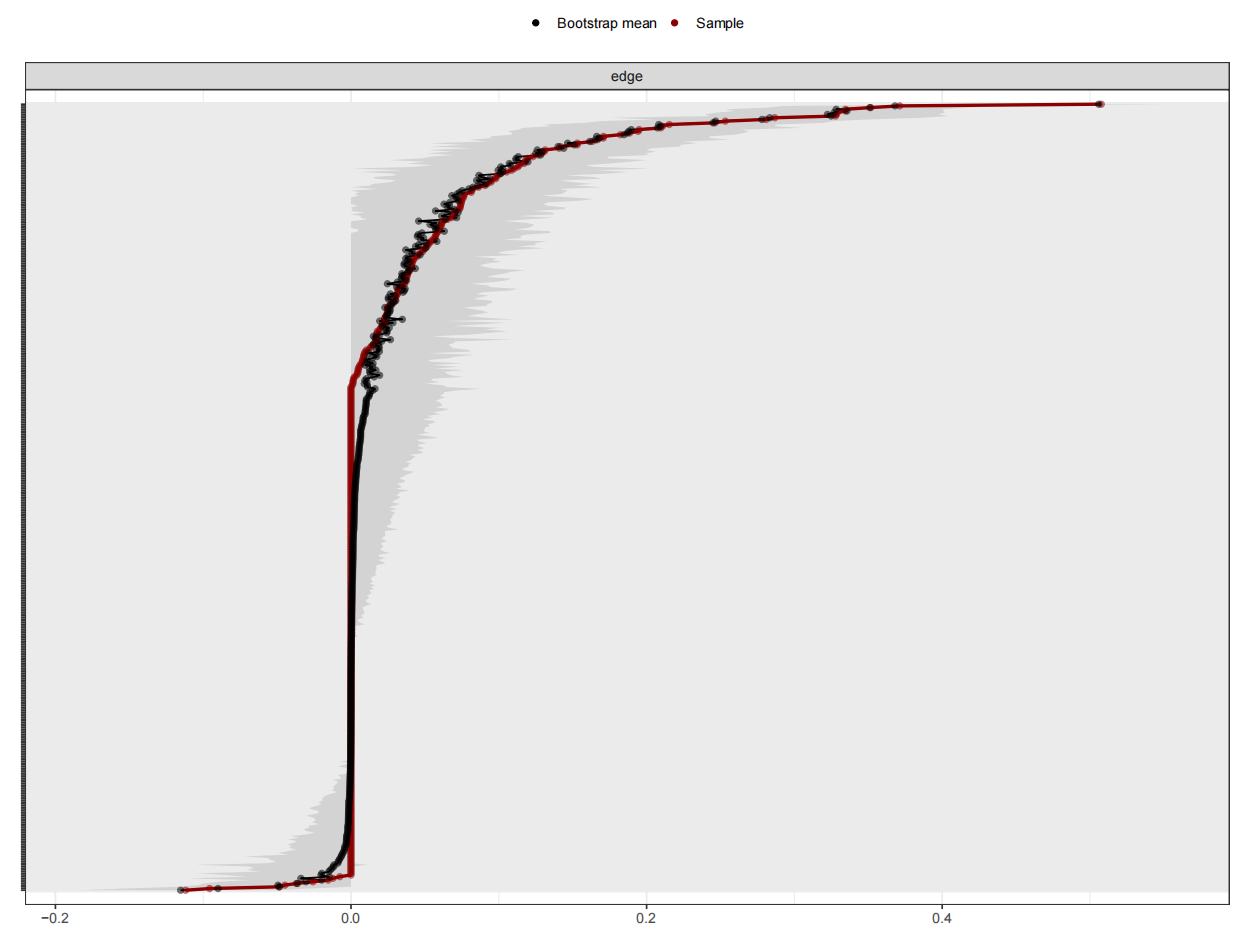


Figure S2. Bootstrapped confidence intervals of estimated edge-weights for the estimated network of 31 nodes. The red line indicates the sample values and the gray area the bootstrapped CIs. Each horizontal line represents one edge of the network. The y-axis labels have been removed to avoid cluttering.


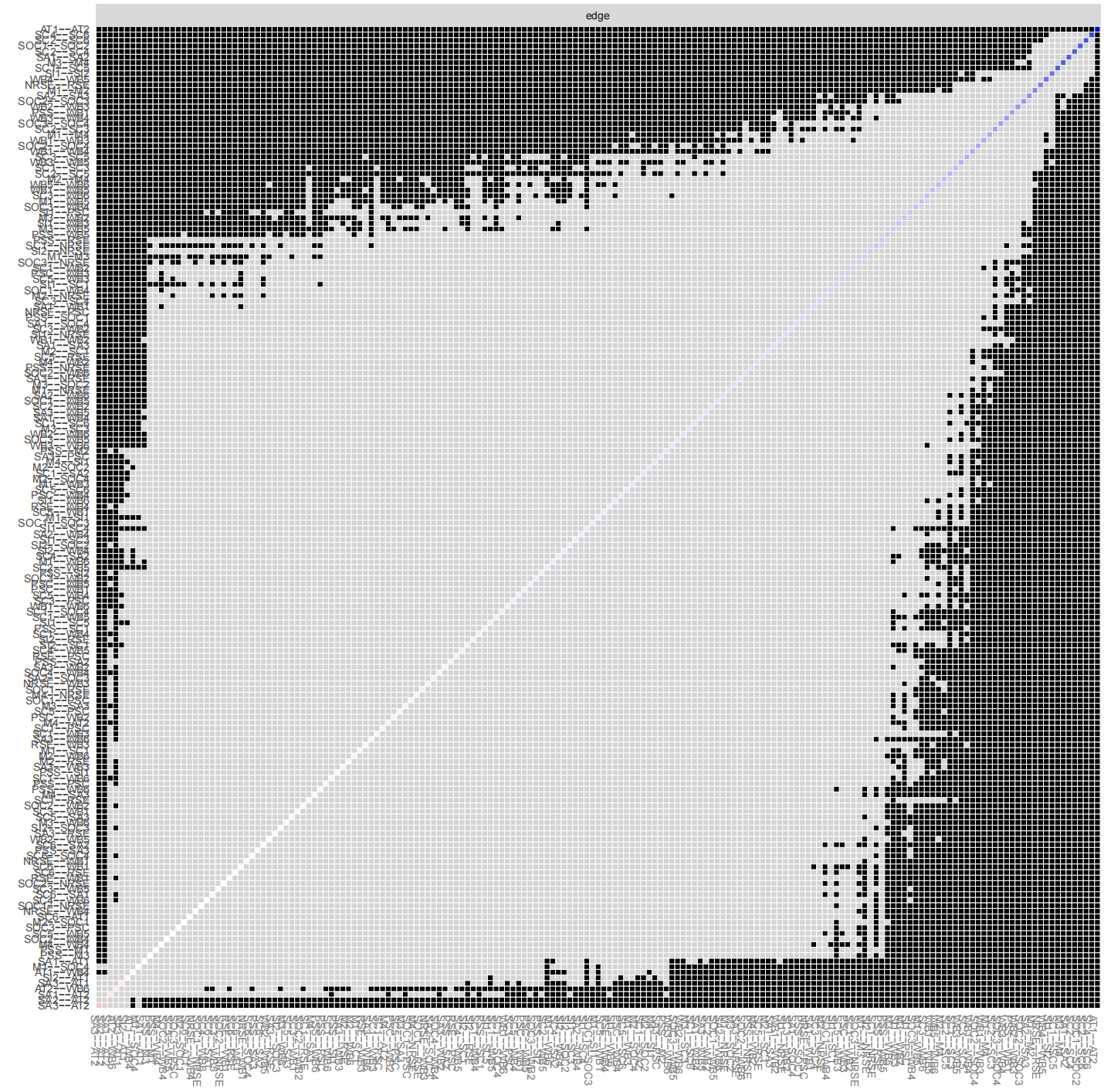


Figure S3. Bootstrapped difference tests (α = 0.05) between edgeweights that were non-zero in the estimated network. Gray boxes indicate edges that do not differ significantly from one-another and black boxes represent edges that do differ significantly from oneanother. Colored boxes in the edge-weight plot correspond to the color of the edge in Fig. 1 in the main text.


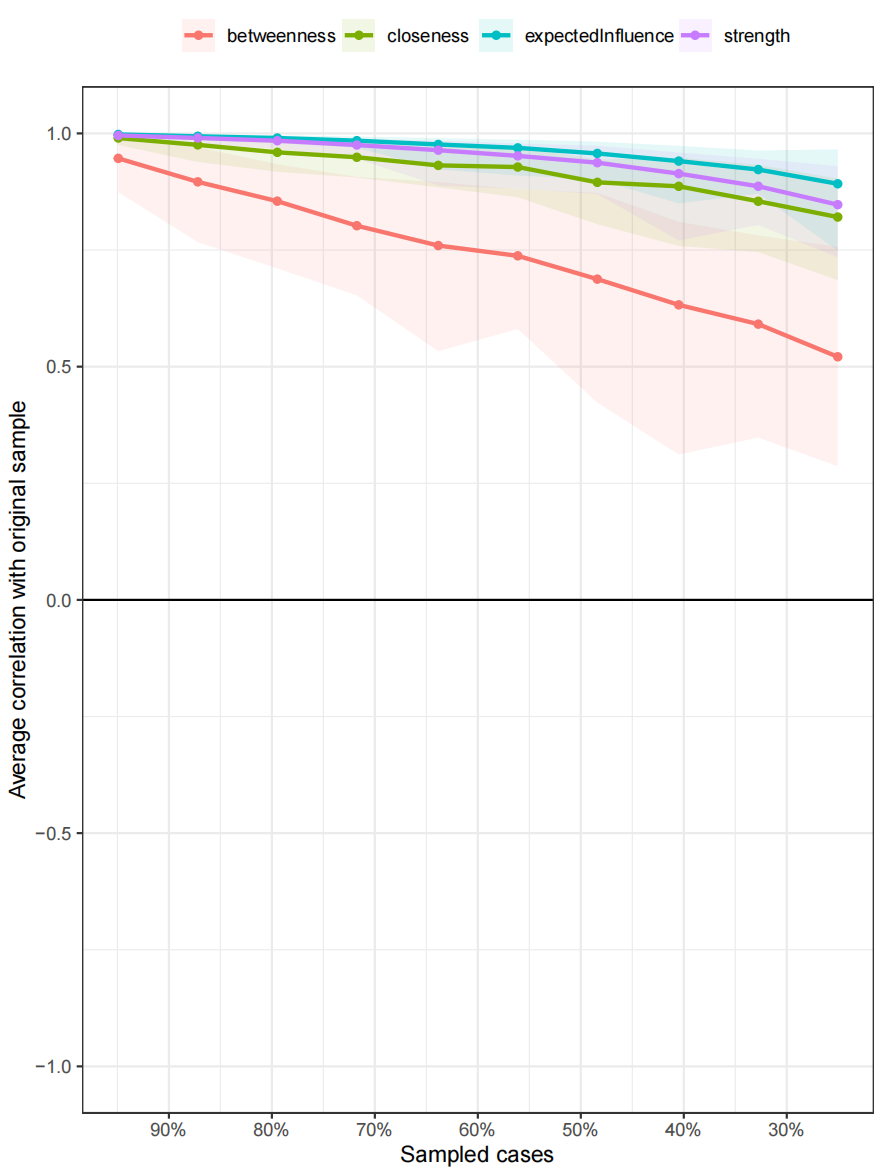


Figure S4. Average correlations between centrality indices of networks sampled with persons dropped and the original sample. Lines indicate the means and areas indicate the range from the 2.5th quantile to the 97.5th quantile.


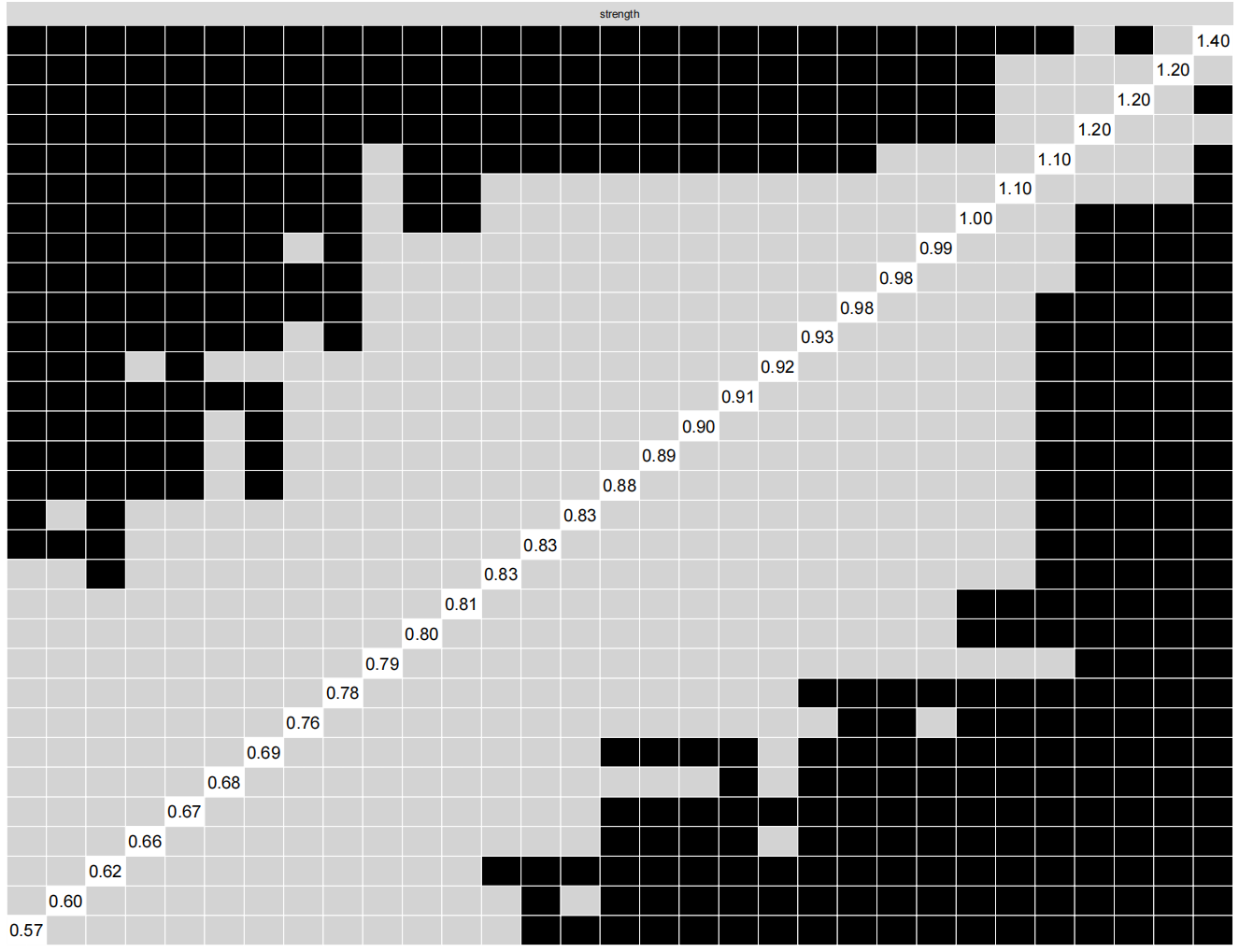


Figure S5. Bootstrapped difference tests (α = 0.05) between node strength of the 31 psychological well-being nodes. Gray boxes indicate nodes that do not differ significantly from one-another and black boxes represent nodes that do differ significantly from oneanother. White boxes in the centrality plot show the value of node strength
